# Complete Genome Sequence of *Lactococcus piscium* CNCM I-4031, a Bioprotective Strain for Seafood Products

**DOI:** 10.1128/genomeA.01510-16

**Published:** 2017-01-26

**Authors:** Laurent Marché, Taous Saraoui, Benoit Remenant, Monique Zagorec, Hervé Prévost, Christine Delbarre-Ladrat, Françoise Leroi, Marie France Pilet

**Affiliations:** a UMR1014 SECALIM, INRA, Oniris, Nantes, France; b Ifremer, Laboratoire Ecosystèmes Microbiens et Molécules Marines pour les Biotechnologies (EM^3^B), Nantes, France

## Abstract

*Lactococcus piscium* CNCM I-4031 is a psychotrophic foodborne lactic acid bacterium showing potential interest for the biopreservation of seafood products due to its inhibition properties toward pathogenic and spoilage bacteria. The analysis of its genome will provide a better understanding of the mechanisms of interaction between these bacteria.

## GENOME ANNOUNCEMENT

*Lactococcus piscium* is a psychrotrophic species first isolated in 1990 from rainbow trout (1). Studies have reported the presence of this species in a large variety of food products, including meat, seafood, and vegetables (2, 3). *L. piscium* CNCM I-4031 (also named EU2241), isolated from raw salmon packs (4), might present interests for seafood biopreservation: it improves the sensory quality of seafood and limits the growth of spoilage (*Brochothrix thermosphacta* and *Serratia proteamaculans*) or pathogenic (*Listeria monocytogenes*) bacteria (5–7). Although the mechanisms of growth inhibition have not yet been elucidated, a previous study suggested the requirement for a contact between *L. piscium* CNCM I-4031 and the target-inhibited strain as well as putative involvement of quorum sensing (8).

The whole-genome sequencing of *L. piscium* CNCM I-4031 was carried out by Eurofins MWG Operon laboratories (Ebersberg, Germany) using GS FLX/FLX+ technologies with shotgun and 8-kbp-long paired-end libraries. The genome sequencing coverage is 63×. The assembly realized with Newbler 2.6 (9) showed an *N*_50_ of 144,750 bp for 31 contigs. The final assembly gave two scaffolds with seven contigs and a genome size of 2.26 Mbp, in accordance with results obtained by pulsed-field gel electrophoresis (PFGE) (data not shown). The first scaffold (2.06 Mb), composed of six contigs, is the complete sequence of the chromosome, with a G+C content of 38.74%. The second scaffold (one contig) formed a 20-kb plasmid with a G+C content of 34.36%. Automatic annotation and manual curation were performed on the whole genome thanks to the MicroScope platform (10, 11) (Genoscope, Évry, France). The results showed that *L. piscium* CNCM I-4031 chromosome contains 2,205 predicted coding DNA sequences (CDSs), as observed in *Lactococcus lactis* genomes (around 2,500).

Thirty-one percent of the predicted CDSs were assigned to proteins of unknown functions, 40% were predicted as enzymes, 6% as transcriptional factors, and 4% as proteins of cell structure. The remaining proteins are involved in diverse cell processes (including carriers, lipoproteins, receptors, and other membrane proteins) or from an extrachromosomal origin. Seventy-five genes (3.4%) were manually curated as carbohydrate-active enzymes (CAZymes) that degrade or modify carbohydrates or create glycosidic bonds.

The plasmid encompasses 26 CDSs, including a complete ribose gene cluster, *rbsRKDACB*. The *rbs* genes are involved in ribose utilization but have also been reported to be responsible for internalization of autoinducer II (AI-2) involved in quorum sensing (12, 13).

The comparison of *L. piscium* CNCM I-4031 genome with that of *L. piscium* MKFS47, a spoiler strain of meat products (14), revealed 384 *L. piscium* CNCM I-4031-specific genes.

### Accession number(s).

This whole-genome shotgun project has been deposited in ENA under the accession no. FLZT01000001 to FLZT01000007. The versions described in this paper are the first versions.

## References

[1] Williams AM, Fryer JL, Collins MD 1990 *Lactococcus piscium* sp. nov. a new *Lactococcus* species from salmonid fish. FEMS Microbiol Lett 68:109–113. doi:10.1111/j.1574-6968.1990.tb04132.x. 1692000

[2] Sakala RM, Hayashidani H, Kato Y, Kaneuchi C, Ogawa M 2002 Isolation and characterization of *Lactococcus piscium* strains from vacuum-packaged refrigerated beef. J Appl Microbiol 92:173–179. doi:10.1046/j.1365-2672.2002.01513.x. 11849342

[3] Vihavainen E, Lundström HS, Susiluoto T, Koort J, Paulin L, Auvinen P, Björkroth KJ 2007 Role of broiler carcasses and processing plant air in contamination of modified-atmosphere-packaged broiler products with psychrotrophic lactic acid bacteria. Appl Environ Microbiol 73:1136–1145. doi:10.1128/AEM.01644-06. 17142357 PMC1828681

[4] Matamoros S, Pilet MF, Gigout F, Prévost H, Leroi F 2009 Selection and evaluation of seafood-borne psychrotrophic lactic acid bacteria as inhibitors of pathogenic and spoilage bacteria. Food Microbiol 26:638–644. doi:10.1016/j.fm.2009.04.011. 19527840

[5] Fall PA, Pilet MF, Leduc F, Cardinal M, Duflos G, Guérin C, Joffraud JJ, Leroi F 2012 Sensory and physicochemical evolution of tropical cooked peeled shrimp inoculated by *Brochothrix thermosphacta* and *Lactococcus piscium* CNCM I-4031 during storage at 8°C. Int J Food Microbiol 152:82–90. doi:10.1016/j.ijfoodmicro.2011.07.015. 21835482

[6] Leroi F, Cornet J, Chevalier F, Cardinal M, Coeuret G, Chaillou S, Joffraud JJ 2015 Selection of bioprotective cultures for preventing cold-smoked salmon spoilage. Int J Food Microbiol 213:79–87. doi:10.1016/j.ijfoodmicro.2015.05.005. 26044337

[7] Fall PA, Leroi F, Chevalier F, Guérin C, Pilet M-F 2010 Protective effect of a non-bacteriocinogenic *Lactococcus piscium* CNCM I-4031 strain against *Listeria monocytogenes* in sterilized tropical cooked peeled shrimp. J Aquat Food Prod Technol 19:84–92. doi:10.1080/10498850.2010.486910.

[8] Saraoui T, Fall PA, Leroi F, Antignac JP, Chéreau S, Pilet MF 2016 Inhibition mechanism of *Listeria monocytogenes* by a bioprotective bacteria *Lactococcus piscium* CNCM I-4031. Food Microbiol 53:70–78. doi:10.1016/j.fm.2015.01.002. 26611171

[9] Zerbino DR, Birney E 2008 Velvet: algorithms for *de novo* short read assembly using de Bruijn graphs. Genome Res 18:821–829. doi:10.1101/gr.074492.107. 18349386 PMC2336801

[10] Vallenet D, Belda E, Calteau A, Cruveiller S, Engelen S, Lajus A, Le Fèvre F, Longin C, Mornico D, Roche D, Rouy Z, Salvignol G, Scarpelli C, Thil Smith AA, Weiman M, Médigue C 2013 MicroScope—an integrated microbial resource for the curation and comparative analysis of genomic and metabolic data. Nucleic Acids Res 41:D636–D647. doi:10.1093/nar/gks1194. 23193269 PMC3531135

[11] Vallenet D, Labarre L, Rouy Z, Barbe V, Bocs S, Cruveiller S, Lajus A, Pascal G, Scarpelli C, Médigue C 2006 MaGe: a microbial genome annotation system supported by synteny results. Nucleic Acids Res 34:53–65. doi:10.1093/nar/gkj406. 16407324 PMC1326237

[12] Taga ME, Semmelhack JL, Bassler BL 2001 The LuxS-dependent autoinducer AI-2 controls the expression of an ABC transporter that functions in AI-2 uptake in *Salmonella* Typhimurium. Mol Microbiol 42:777–793. doi:10.1046/j.1365-2958.2001.02669.x. 11722742

[13] Armbruster CE, Pang B, Murrah K, Juneau RA, Perez AC, Weimer KED, Swords WE 2011 RbsB (NTHI_0632) mediates quorum signal uptake in nontypeable *Haemophilus influenzae* strain 86–028NP. Mol Microbiol 82:836–850. doi:10.1111/j.1365-2958.2011.07831.x. 21923771 PMC3609414

[14] Andreevskaya M, Johansson P, Laine P, Smolander OP, Sonck M, Rahkila R, Jääskeläinen E, Paulin L, Auvinen P, Björkroth J 2015 Genome sequence and transcriptome analysis of meat spoilage lactic acid bacterium *Lactococcus piscium* MKFS47. Appl Environ Microbiol 81:3800–3811. doi:10.1128/AEM.00320-15. 25819958 PMC4421071

